# Neo-epitopes emerging in the degenerative hippocampal granules of aged mice can be recognized by natural IgM auto-antibodies

**DOI:** 10.1186/s12979-015-0050-z

**Published:** 2015-11-24

**Authors:** Gemma Manich, Elisabet Augé, Itsaso Cabezón, Mercè Pallàs, Jordi Vilaplana, Carme Pelegrí

**Affiliations:** Departament de Fisiologia, Facultat de Farmàcia, Universitat de Barcelona, Av. Joan XXIII s/n, 08028 Barcelona, Spain; Unitat de Farmacologia i Farmacognòsia, Facultat de Farmàcia, Institut de Biomedicina (IBUB), Universitat de Barcelona, Av. Joan XXIII s/n, 08028 Barcelona, Spain; CIBERNED Centros de Biomedicina en Red de Enfermedades Neurodegenerativas, Barcelona, Spain

**Keywords:** Ageing, Hippocampus, Auto-antibody, IgM, Neo-epitope, Periodic acid-Schiff

## Abstract

**Background:**

Degenerative granular structures appear progressively with age in the hippocampus of most mouse strains. We recently reported that these granules contain a neo-epitope that is recognised by IgM antibodies present as contaminants in many commercial antibodies obtained from mouse ascites and mouse or rabbit serum. We hypothesise that these anti-neo-epitope IgMs are in fact natural auto-antibodies that are generated spontaneously during the foetal stage without previous contact with external antigens and whose repertoire and reactivity pattern have been determined through evolution, being remarkably stable within species and even between species.

**Findings:**

In the present work we found that mice from the ICR-CD1, BALB/C and SAMP8 strains have anti-neo-epitope IgM antibodies in their plasma at all ages tested and even when maintained under specific opportunistic pathogen-free conditions. Moreover, we determined that these anti-neo-epitope IgMs are also present in rabbit, goat and rat serum. We also found that, in each mouse that presented hippocampal granules, the anti-neo-epitope IgMs contained in its plasma recognised the neo-epitopes in its own granules.

**Conclusions:**

This study led to the conclusion that anti-neo-epitope IgMs are widespread natural auto-antibodies contained in the plasma of mice and other species. The presence of these natural auto-antibodies not only explains why they are frequently found as contaminants in commercial antibodies, but also paves the way for a new approach to a treatment and diagnosis of pathological brain processes based on natural IgMs and neo-epitopes.

## Findings

### Introduction

Pathological granular structures, originally described as periodic acid-Schiff granules due to their reactivity to this staining, appear progressively with age in the hippocampus of most mouse strains, especially in the senescence-accelerated mouse prone 8 (SAMP8) strain [1–4]. They are round-to-ovoid structures that measure up to 3 μm in diameter and tend to form clusters, each with approximately 40–50 granules [1, 4, 5]. The ultrastructural analysis of the granules showed a central electron-dense core formed by an accumulation of membranous fragments and a peripheral zone, or halo, externally delimited by a slightly discontinuous plasma membrane. The peripheral zone contains degenerating organelles, especially mitochondria, and unstable membranous structures that lead to the formation of blebs or bubbles, which cover an extensive area of this zone [2, 5–7]. In recent studies, we observed that these granules are formed by degenerative mechanisms that occur mainly in astrocytic processes, with each affected astrocyte generating one cluster of granules [8]. This degenerative process can also affect the structures of the surrounding neuropil, including neuronal structures such as synaptic buttons and dendritic spines [8]. In another recent study, we reported the presence of specific epitopes in the granules that are not present in healthy brain structures and are formed at the same time as the granules, which led us to conclude that they were neo-epitopes [9]. These neo-epitopes are mainly located in the membranous fragments of the centre of the granules, although during the granule formation they can also be found in the membranes of the adjacent neuropil. Moreover, we showed that this neo-epitope is recognised by contaminant IgM antibodies present in many commercial antibodies obtained from mouse ascites and mouse or rabbit serum.

All these findings suggest that the contaminant IgMs found in antibodies obtained from mouse serum and ascites and directed against the neo-epitope could in fact be natural auto-antibodies. Natural antibodies are generated spontaneously from the foetal stage without previous contact with external antigens [10, 11]. Their repertoire and reactivity pattern have been determined through evolution and are remarkably stable within species and even between species [12, 13]. They constitute a first line of immune defence and also have other important functions in the physiology of the organism. Some of these natural antibodies are able to recognise the neo-epitopes that are formed, for example, in cell remnants or in apoptotic or altered cells, and thus intervene in their controlled elimination [12]. Consequently, we hypothesise that mouse plasma contains natural IgM auto-antibodies that recognise the neo-epitopes present in the degenerative hippocampal granular structures formed with ageing.

If our hypothesis is correct and the anti-neo-epitope IgMs are natural antibodies, all mice of the different strains should have anti-neo-epitope IgMs in their blood plasma at all ages, regardless of any previous exposure to external pathogens, or whether the animals develop pathological granular structures in their hippocampus or not. Moreover, they are probably also found in the plasma of mammals other than mice. Besides, in order to consider them as auto-antibodies, it is necessary to demonstrate that the anti-neo-epitope IgMs in the plasma of animals with granules recognise the neo-epitopes in their own granules.

## Methods

### Mice

Male SAMP8, ICR-CD1 and Balb/cOlaHsd (BALB/C) mice aged from 1 to 15-month were used. These strains were selected because their progressive formation of hippocampal granule clusters is accelerated, moderate or nearly absent, respectively. ICR-CD1 and SAMP8 mice were obtained from the Servei d’Estabulari de la Facultat de Farmàcia (Universitat de Barcelona), while BALB/C mice were obtained from Harlan Laboratories Models (Sant Feliu de Codines, Spain). Animals were kept under standard temperature conditions (22 ± 2 °C) and 12:12 h light–dark cycles (300 lux / 0 lux), and had access to food and water *ad libitum* throughout the study. We also used 3-month-old male ICR-CD1 (Janvier Labs, Le Genest-Saint-Isle, France) and BALB/C mice (Harlan Laboratories Models), which were kept under specific opportunistic pathogen-free (SOPF) conditions from birth and until the day of sacrifice. All experimental procedures were reviewed and approved by the University of Barcelona Animal Experimentation Ethics Committee (DAAM 7504).

### Serum obtention and brain removal and processing

One to nine-months-old SAMP8, ICR-CD1 and BALB/C mice (4–6 animals per group) were anaesthetised i.p. with sodium pentobarbital (80 mg/kg), the thoracic cage was opened and blood was obtained by cardiac puncture. Blood was left to clot, centrifuged at 14,850 rpm for 5 min in a Biofuge Pico centrifuge (Heraeus, Madrid, Spain), and serum was collected and stored at −80 °C until use. After cardiac puncture, animals received an intracardiac gravity-dependent perfusion of 50 mL saline solution (0.9 % NaCl). Brains were dissected and cryostatic brain sections were obtained as described previously [8]. Some brain sections from 12 and 15-month-old ICR-CD1 mice were obtained following the same procedure, but without the cardiac puncture to obtain blood, in order to obtain hipocampal sections with degenerative granules.

### Immunohistochemical staining for fluorescence microscopy and image acquisition

In order to determine if the plasma of the animals (including those born and maintained in SOPF conditions) contained anti-neo-epitope IgMs, immunohistochemical staining procedures were performed on brain sections from 12 or 15-months-old ICR-CD1 animals by using the mouse serum in the first incubation and an anti-mouse IgM conjugated to a fluorocrom as a secondary antibody. The staining was performed as follows. Sections were rehydrated with PBS and then blocked and permeabilised with 1 % bovine serum albumin (Sigma-Aldrich) and 0.1 % Triton X-100 (Sigma-Aldrich) in PBS for 20 min. They were washed with PBS and the primary incubation was performed overnight at 4 °C with a solution containing both a mouse serum diluted 1/100 and the rabbit anti-MMP2 IgG antibody (Merck Millipore, Darmstadt, Germany) diluted 1/200 in blocking buffer (BB; 1 % bovine serum albumin in PBS). The anti-MMP2 antibody was added as a specific marker of the granules, as we previously described [9]. Thereafter, slides were washed again and incubated for 1 h with a solution containing 1/50 tetramethyl rhodamine-conjugated (TRITC) goat anti-mouse IgM (Jackson ImmunoResearch Laboratories, Newmarket, UK) and 1/250 AlexaFluor (AF)-488-conjugated donkey anti-rabbit IgG antibody (Life Technologies, Carlsbad, CA) in BB. Nuclear staining was performed with Hoechst (2 μg/mL, H-33258, Fluka, Madrid, Spain). The slides were coverslipped with Fluoromount (Electron Microscopy Sciences, Hatfield, PA, USA). As the positive control for the anti-neo-epitope IgM staining of the granules the anti-neo-epitope IgMs contained as a contaminant in the OX52 antibody (obtained from mouse ascites) were used instead of the animal serum. Negative controls were performed with a first incubation without mouse serum. To verify that the anti-neo-epitope IgMs contained in mouse serum were auto-antibodies, we performed the same immunohistochemical procedure detailed above but on brain sections of the same animal that provided the serum instead of the brain sections from aged ICR-CD1 animals.

To determine the presence of the anti-neo-epitope IgMs in the serum of other species than mouse we performed the same immunohistochemical staining procedures by using rat serum (obtained from our laboratory), rabbit serum and goat serum (both from Jackson ImmunoResearch Laboratories) at a 1/50 dilution instead of mouse serum. AF-594-conjugated goat anti-rat IgM (Life Technologies), fluorescein isothiocyanate (FITC)-conjugated goat anti-rabbit IgM, FITC-conjugated rabbit anti-goat IgM (Abcam, Cambridge, UK) and AF-555-conjugated donkey anti-rabbit IgG (Life Technologies) were used at a dilution 1/250 in the secondary incubations.

In all cases, fluorescence images were taken with a fluorescence laser microscope BX41 (Olympus, Germany) and images where stored in tiff format. Image compositions were performed with the ImageJ programme (National Institute of Health, USA).

## Results

**Fig. 1:**
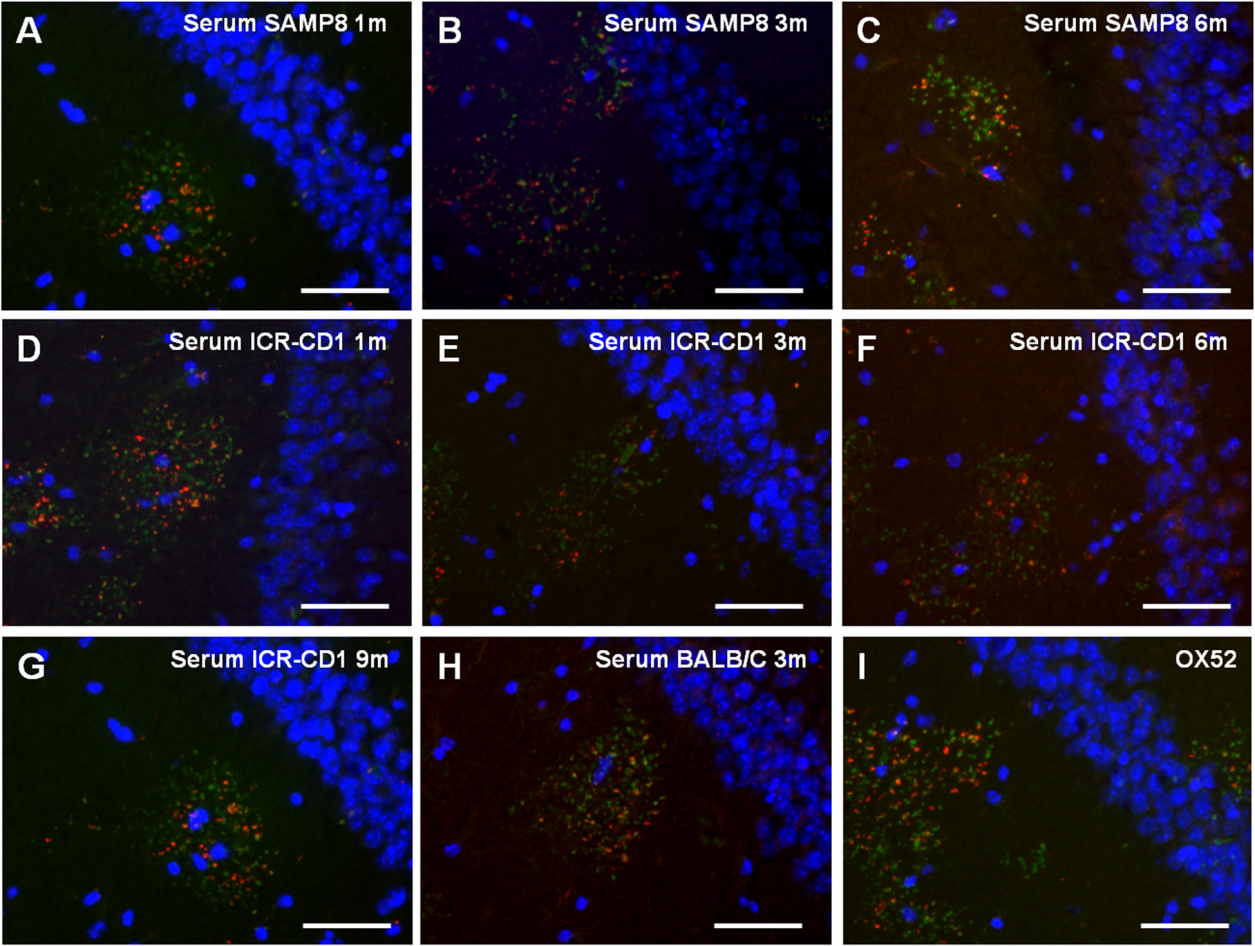
Sera from SAMP8, ICR-CD1 and BALB/C mice contain anti-neo-epitope IgMs. Representative images of the hippocampal region of brain sections from 15-month-old ICR-CD1 mice simultaneously stained with Hoechst (blue), anti-MMP-2 antibody (green) and mouse sera (red) from each experimental group as detailed next. **a**-**c**: Sera from SAMP8 mice aged 1, 3 and 6 months, respectively. **d**-**g**: Sera from ICR-CD1 mice aged 1, 3, 6 and 9 months, respectively. **h**: Serum from a BALB/C mouse aged 3 months. **i**: Control staining of the granules with anti-neo-epitope IgMs contained in the OX52 antibody. In all cases the anti-MMP-2 staining showed the clusters of granules. Some granules of the clusters are stained with the serum, indicating that all sera contained anti-neo-epitope IgMs. Scale bar: 50 μm

In order to determine whether the anti-neo-epitope IgMs are natural antibodies we first examined their presence in the serum of mice from different strains at different ages, i.e., 1, 3, 6 and 9 month-old ICR-CD1 mice, 1, 3 and 6 month-old SAMP8 mice and 3 month-old BALB/C mice (*n* = 4–6 per group). The IgM presence was evaluated by immunostaining brain sections from 15-month-old ICR-CD1 mice with the mouse sera and the anti-MMP2 antibody. In all cases the sera stained hippocampal granules, which are also labelled with the anti-MMP2 antibody, indicating that all tested mouse sera contained the anti-neo-epitope IgMs (Fig. 1a–h). In the positive control performed with the anti-neo-epitope IgMs contained in the OX52 antibody, granules were also stained (Fig. 1i).

**Fig. 2:**
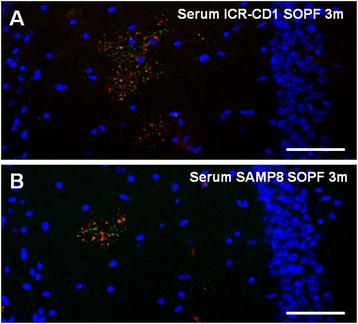
Previous contact with external pathogens is not necessary for the presence of anti-neo-epitope IgMs in mouse serum. Representative images of the hippocampal region of brain sections from 12-month old ICR-CD1 mice simultaneously stained with Hoechst (blue), anti-MMP-2 antibody (green) and sera (red) from a 3-month-old ICR-CD1 SOPF animal (**a**) or from a 3-month-old BALB/C SOPF animal (**b**). In both cases the anti-MMP-2 staining showed the clusters of granules. Some granules of the clusters are stained with the serum, indicating that the sera of SOPF animals contained anti-neo-epitope IgMs. Scale bar: 50 μm

On the other hand, as natural antibodies are present regardless of any previous exposure to external pathogens, we also tested for the presence of the anti-neo-epitope IgMs in the serum of 3-month-old ICR-CD1 and BALB/C mice maintained under SOPF conditions (*n* = 6 per group). All mouse sera positively stained the hippocampal granules contained in the brain sections from 15-month-old ICR-CD1 mice, which are also labelled with the anti-MMP2 antibody (Fig. 2), indicating that mice kept under SOPF conditions contained the anti-neo-epitope IgMs in their plasma.

**Fig. 3:**
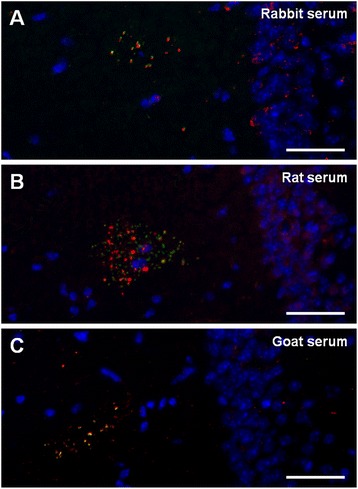
Anti-neo-epitope IgMs are present in the serum from other species than mice. Representative images of the hippocampal region of brain sections from 12-month-old ICR-CD1 mice simultaneously stained with Hoechst (blue), anti-MMP-2 antibody and rabbit (**a**), rat (**b**) and goat (**c**) serum. Clusters of granules can be observed with the anti-MMP-2 staining (red in **a** and **c** and green in **b**) and some granules of the clusters are stained with the different sera (green in **a** and **c** and red in **b**), which indicates that the serum of these species contained anti-neo-epitope IgMs. Scale bar: 50 μm

Furthermore, as natural antibodies are remarkably stable not only within species but even between species, we also tested the presence of these IgMs in the serum of species other than mice. Thus, we immunostained brain sections of a 12-month old ICR-CD1 mouse with rat, rabbit and goat serum. In all cases the granules were positively stained, which indicated that the serum of the different species assayed contained the anti-neo-epitope IgMs (Fig. 3).

**Fig. 4:**
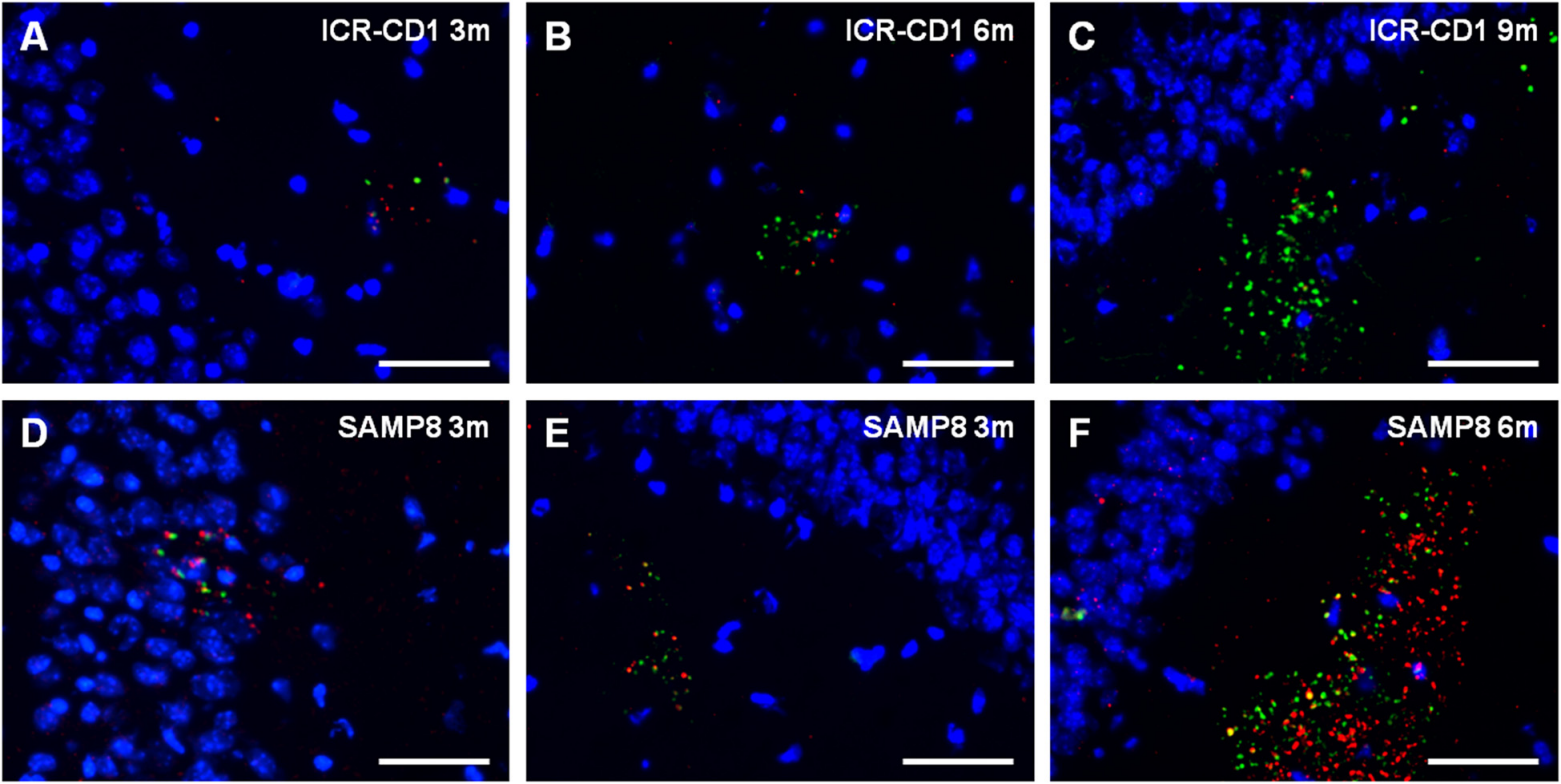
Anti-neo-epitope IgMs are auto-antibodies. Representative images of the hippocampal region of brain sections from different mice stained with their own serum (*red*) and both the anti-MMP2 antibody (*green*) and Hoechst (*blue*). **a**-**c**: Representative ICR-CD1 mice aged 3, 6 and 9 months, respectively. **d**-**f**: Representative SAMP8 mice aged 3 and 6 months. As can be observed, the granules are stained by the serum of the own mouse. Thus, each serum recognize the own neo-epitope present in its clusters of granules. Scale bar: 50 μm

On the other hand, in order to establish whether the anti-neo-epitope IgM is an auto-antibody, brain sections from each mouse were immunostained with its own sera combined with the staining with the anti-MMP2 antibody. In the cases in which MMP2 indicated the presence of granules, the granules were also stained with the own serum, indicating that the anti-neo-epitope IgM contained in serum is an auto-antibody that recognises the own hippocampal granules (Fig. 4). It must be pointed out that some young animals, especially BALB/C mice, do not present granules in their hippocampus and thus the staining of the granules with their own IgMs cannot be tested. Moreover, as all sera from all ages contain anti-neo-epitope IgMs, these results also indicate that the presence of the granules in the brain is not necessary for the existence of the anti-neo-epitope IgMs in the plasma.

**Fig. 5:**
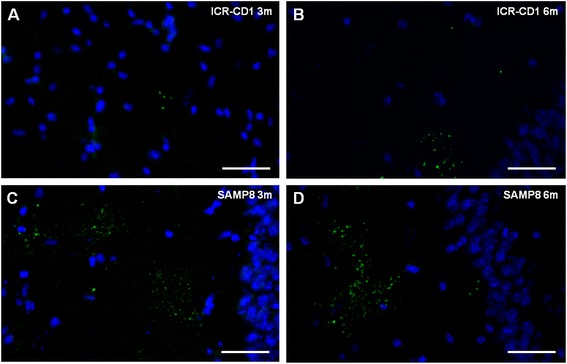
IgMs from mouse plasma do not reach the granules of the hippocampus under physiological conditions. Representative images of the hippocampal region of brain sections from different mice stained with both the anti-MMP2 antibody (*green*) and Hoechst (*blue*). In the second incubation, an anti-mouse IgM antibody *(red)* is added. **a** and **b**: brain sections from representative ICR-CD1 mice aged 3 and 6 months, respectively. **c** and **d**: brain sections from representative SAMP8 mice aged 3 and 6 months, respectively. The granules are stained with the MMP2 antibody but the secondary anti-mouse IgM antibody did not stain the granules in any of the assayed mice, thus indicating that the anti-neo-epitope IgMs contained in their plasma do not reach the granules. Scale bar: 50 μm

Moreover, it should be pointed out that, under physiological conditions, the IgMs present in mouse plasma do not reach the granules of the hippocampus. The immunostaining with a primary incubation containing only the antibody directed against MMP2 and a secondary incubation with antibodies directed against the anti-MMP2 and against the mouse IgMs show the clusters of granules stained with the anti-MMP2 antibody but not with the anti-IgM antibody (Fig. 5). Thus, the anti-neoepitope IgMs existing in the plasma of the animals do not have access to the brain neo-epitopes.

## Discussion and conclusions

The results of this study led us to conclude that the IgMs directed against the neo-epitope are common natural auto-antibodies that are always contained in mouse plasma independently of the existence of granules in the brain, and are also present in plasma of species other than mouse.

The presence of both these neo-epitopes in the granules and the natural anti-neo-epitope IgMs could be due to the fact that the neo-epitopes are formed by widespread mechanisms related to pathological ageing processes such as those associated with the increase of oxidative stress caused by the production of reactive oxygen or nitrogen species. Note that the number of granules seems to be modulated by antioxidant treatments or increases in oxidative stress [5, 14–16]. Oxidative stress may cause advanced glycation end products or oxidised phospholipids, which are a target of natural auto-antibodies and may trigger immune responses aimed at eliminating them and removing the structures that contain them [17–19].

Oxidation-specific epitopes have been documented in some pathological situations as in atherosclerotic lesions but also in pulmonary, renal and liver diseases as well as central nervous system lesions of multiple sclerosis and Alzheimer disease. Moreover, cells undergoing apoptosis have also been shown to contain multiple oxidation-specific epitopes in their plasma membranes. Therefore, oxidation-specific epitopes constitute perfect tags for the identification of biological waste and the discrimination of dying cells from viable cells by specific immune responses [20]. From this point of view, we can consider that the neo-epitopes found in the granules of the hippocampus of aged mice and the presence of natural anti-neo-epitope IgMs in their plasma could be related to a general mechanism that is effective in some tissues. However, the brain avoids these responses, probably due to the existence of the blood–brain barrier. It is worth noting that natural IgMs and neo-epitopes that have received little attention are now back on stage, as demonstrated by a new field of activity relating to the diagnosis and treatment of some cancers based on the presence of specific tumour neo-epitopes, which can be recognised by natural IgMs [21–23]. The results of this study led us to believe that treatment and diagnostics based on natural IgMs and neo-epitopes can probably be extended to brain diseases and pathological processes that occur in the brain, and could therefore pave the way for a new field of study in this area.

## Abbreviations

BB: blocking buffer
FITC: fluorescein isothiocyanate-conjugated
IgM: immunoglobulin M
PBS: phosphate-buffered saline
SAMP8: senescence-accelerated mouse prone 8
SOPF: specific opportunistic pathogen free
TRITC: tetramethyl rhodamine-conjugated
