# Correction: Neo-epitopes emerging in the degenerative hippocampal granules of aged mice can be recognized by natural IgM auto-antibodies

**DOI:** 10.1186/s12979-022-00293-w

**Published:** 2022-08-15

**Authors:** Gemma Manich, Elisabet Augé, Itsaso Cabezón, Mercè Pallàs, Jordi Vilaplana, Carme Pelegrí

**Affiliations:** 1 Departament de Fisiologia, Facultat de Farmàcia, Universitat de Barcelona, Av. Joan XXIII s/n, 08028 Barcelona, Spain; 2 Unitat de Farmacologia I Farmacognòsia, Facultat de Farmàcia, Institut de Biomedicina (IBUB), Universitat de Barcelona, Av. Joan XXIII s/n, 08028 Barcelona, Spain; 3 CIBERNED Centros de Biomedicina en Red de Enfermedades Neurodegenerativas, Barcelona, Spain

**Correction: Immun Ageing 12, 23 (2015)**

**https://doi.org/10.1186/s12979-015-0050-z**

Following publication of the original article [1], the authors identified an error in Fig. 1. All the images shown in Fig. 1 (from image A to I) are crops of larger images. To obtain the image G, we mistakenly used the source of the image A instead of the source of the image G. In this new version, the image G has been corrected. The legend of the image remains correct. The correct figure is given below.

**Fig. 1:**
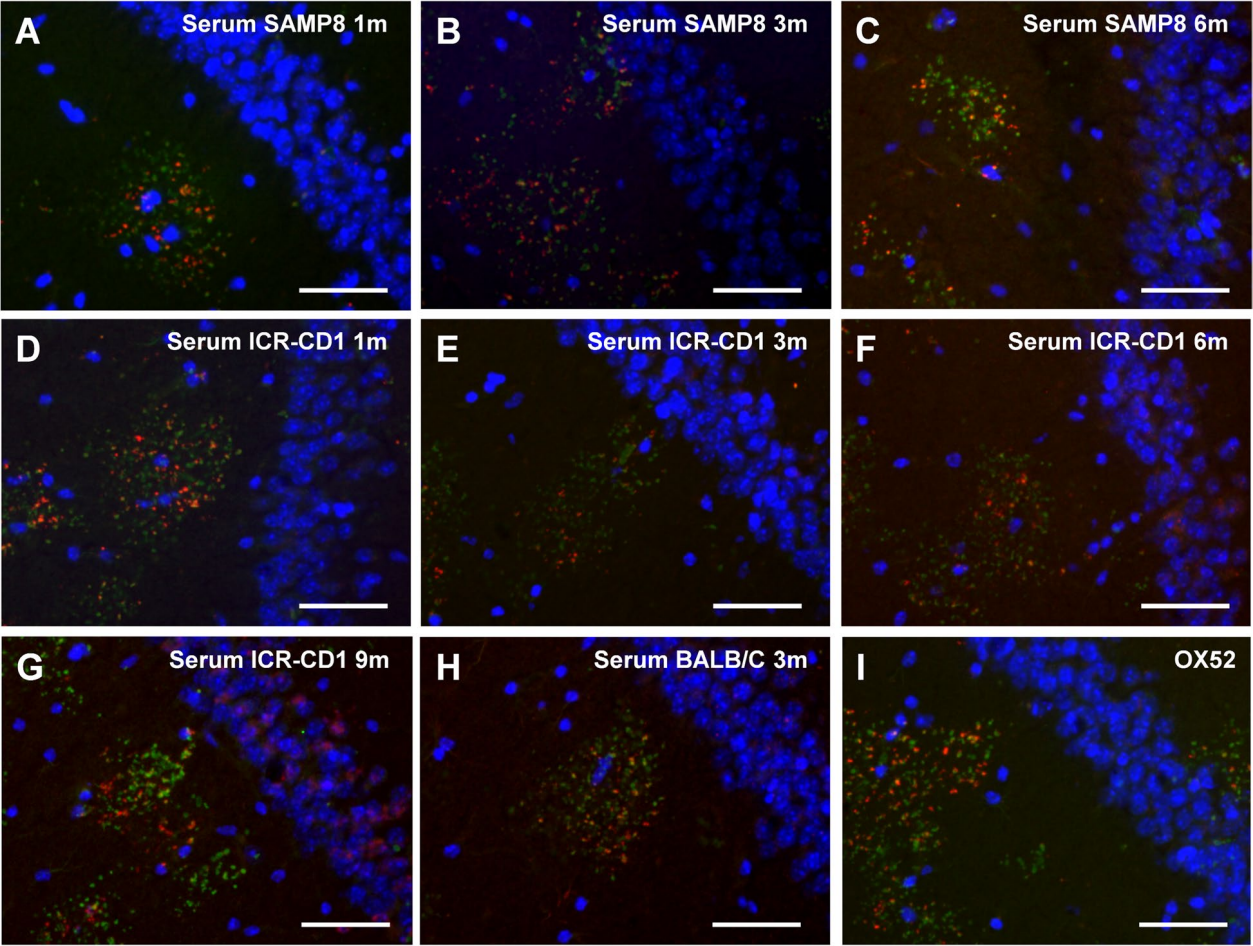
Sera from SAMP8, ICR-CD1 and BALB/C mice contain anti-neo-epitope IgMs. Representative images of the hippocampal region of brain sections from 15-month-old ICR-CD1 mice simultaneously stained with Hoechst (blue), anti-MMP-2 antibody (green) and mouse sera (red) from each experimental group as detailed next. **a**-**c**: Sera from SAMP8 mice aged 1, 3 and 6 months, respectively. **d**-**g**: Sera from ICR-CD1 mice aged 1, 3, 6 and 9 months, respectively. **h**: Serum from a BALB/C mouse aged 3 months. **i**: Control staining of the granules with anti-neo-epitope IgMs contained in the OX52 antibody. In all cases the anti-MMP-2 staining showed the clusters of granules. Some granules of the clusters are stained with the serum, indicating that all sera contained anti-neo-epitope IgMs. Scale bar: 50 μm
